# K-Ar geochronology for hydrothermal K-feldspar within plagioclase in a granitic pluton: constraints on timing and thermal condition for hydrothermal alteration

**DOI:** 10.1016/j.heliyon.2021.e06750

**Published:** 2021-04-09

**Authors:** Takashi Yuguchi, Koshi Yagi, Eiji Sasao, Tadao Nishiyama

**Affiliations:** a Faculty of Science, Yamagata University, 1-4-12 Kojirakawa, Yamagata, 990-8560, Japan; b Hiruzen Institute for Geology and Chronology Co., Ltd., 2-5, Nakashima, Naka-ku, Okayama, 703-8252, Japan; c Japan Atomic Energy Agency, 1-64, Yamanouchi, Akiyo, Mizunami, Gifu, 509-6132, Japan; d Graduate School of Science and Technology, Kumamoto University, 2-39-1, Kurokami, Chuo-ku, Kumamoto, 860-8555, Japan

**Keywords:** K-feldspar K-Ar age, Plagioclase alteration, Solid-state replacement, Dissolution and precipitation, K-feldspathization and illitization, Toki granitic pluton

## Abstract

This study presents the K-Ar geochronology for hydrothermal K-feldspar in plagioclase alteration, including methodology and application to the Toki granite, in central Japan. Borehole samples from the Toki granite were collected and mechanically and chemically processed to separate plagioclase from the rock and remove bulk impurities. The sample fraction of cleaned plagioclase powder was further processed to a smaller size fraction, allowing separation of the altered K-feldspar from the plagioclase host. The resulting K-feldspar represented the hydrothermal alteration product and was characterized crystallographically as microcline, and its K-Ar ages were measured. The results of the K-Ar dating and petrographic characterization indicated that in this setting, plagioclase alteration occurred through a combination of solid-state replacement and dissolution–precipitation processes. The K-feldspathization age enables constraint of the temporal conditions of the solid-state replacement process to 62.2 ± 1.4 Ma. The time-temperature (*t-T*) path of the sampling site is an effective tool for determining both the timing and thermal conditions of the hydrothermal microcline formation in plagioclase alteration. The combination of the *t-T* path and the microcline K-Ar age provides formation temperatures of about 307–325 °C. The timing and thermal conditions of solid-state replacement (62.2 ± 1.4 Ma and 325–307 °C) indicate an older age and a higher temperature than those of dissolution–precipitation (59.2 ± 1.4 Ma and 305–290 °C: Yuguchi et al., 2019A). The plagioclase alteration consists of serial processes from solid-state replacement to dissolution–precipitation. Addition of the thermal conditions and timing into petrography have implications for the sequential phenomenal variation in granite.

## 1 Introduction

**Figure 1:**
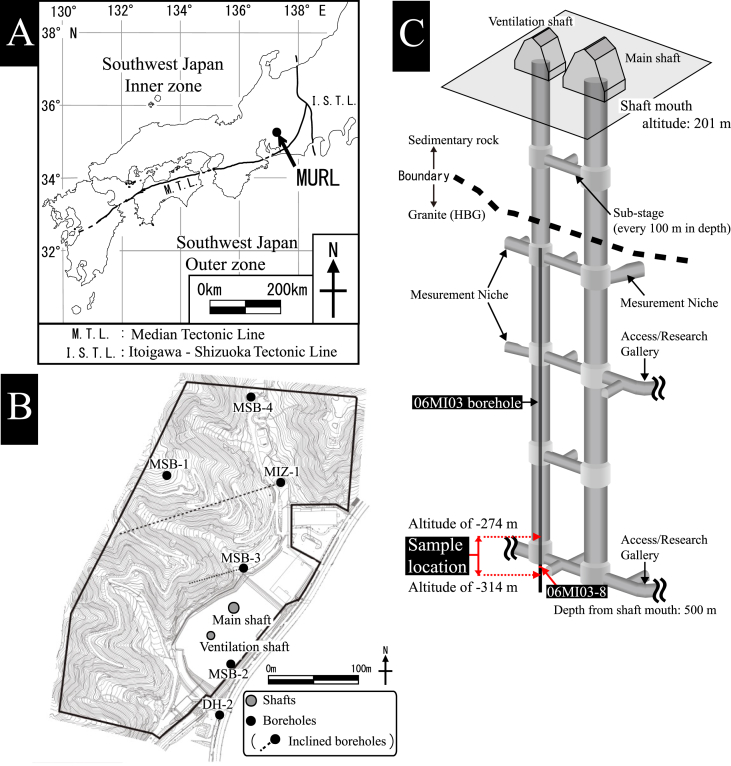
The Toki granite and Mizunami Underground Research Laboratory (MURL). (A) Location map of the MURL. (B) Shafts and boreholes in the Mizunami Underground Research Laboratory. (C) Schematic overview of Mizunami Underground Research Laboratory and the sample locations used in this study. The samples were collected from borehole 06MI03 at an altitude of –304 masl (meters above sea level) in the HBG.

Fluid is vital in hydrothermal reactions (Putnis et al., 2007) because fluid chemistry determines mineral assemblages in hydrothermal alteration. The petrography, timing, and thermal conditions of alteration product minerals reveal the long-term fluid chemistry fluctuations during sub-solidus cooling (Yuguchi et al., 2015, 2019A). Formation ages and temperature determinations for hydrothermal minerals are significant constraints to the timing and thermal conditions of the alteration process by hydrothermal fluids. This study focuses on K-Ar geochronology for hydrothermal K-feldspar in plagioclase alteration, including methodology and application to the Toki granite, central Japan (Figure 1).

Hydrothermal alterations of granitic rocks involve ubiquitous biotite chloritization and plagioclase alteration. Correspondingly, the Toki granite experiences serial alteration from biotite chloritization to plagioclase alteration occurring 68–51 Ma at temperatures of 180–350 °C (Yuguchi et al., 2015, 2019A). The alterations were derived from infiltration of hydrothermal fluid along microcracks and through micropores (Nishimoto et al., 2008; Nishimoto and Yoshida, 2010; Yuguchi et al., 2015, 2019A). Plagioclase alteration involves albitization and K-feldspathization, occurring through solid-state replacement within plagioclase, and illite, calcite, fluorite, and epidote formation, occurring via dissolution of plagioclase around micropores and precipitation into vacancies (Yuguchi et al., 2019A). Thus, the alteration is a combination of solid-state replacement and dissolution–precipitation processes. The illitization age of 59.2 ± 1.4 Ma reflects the dissolution–precipitation process determined by illite K-Ar geochronology (Yuguchi et al., 2019A). The K-feldspathization age enables constraint of the solid-state replacement process timing, and also leads to timing differences between solid-state replacement and dissolution–precipitation. This study presents companion data and follows the paper by Yuguchi et al. (2019A).

There are some problems to be solved in obtaining a “true” K-Ar age for K-feldspar: 1) petrographical demonstration that hydrothermal K-feldspar grains did not experience argon-loss, and 2) separation of hydrothermal K-feldspar grains from the interior of plagioclase. Argon leakage from K-feldspar generally yields a misleading young age, in the K-Ar geochronology system. Petrography in the target sample of the Toki granite demonstrates that the K-feldspar grains had little argon-loss (see petrography), thus, it is ideal for such studies. It is technically difficult to separate the hydrothermal K-feldspar grains for age determination because the fine-grained K-feldspar of hydrothermal origin occurs within the plagioclase (see petrography). This study presents new multistep procedures for separation of plagioclase grains from the rock and for hydrothermal K-feldspar from plagioclase.

The Toki granite has vertical shafts approximately 500 m long, allowing us to collect samples from deep within the pluton. These samples have escaped weathering and are suitable for petrographical study of biotite chloritization (Yuguchi et al., 2015) and plagioclase alteration (Yuguchi et al., 2019A). Yuguchi et al. (2019B) presented the position-specific time-temperature (*t-T*) paths of fifteen samples within the Toki granite. The *t-T* paths of the sampling site are effective tools for determining both the timing and thermal condition of the secondary minerals; the age (or temperature) of the secondary mineral will provide the corresponding temperature (or age) through the *t-T* path. Therefore, the serial alteration phenomena consisting of solid-state replacement and dissolution–precipitation in plagioclase alteration can be established for both timing and thermal condition. The K-feldspar closure temperature cannot be determined without diffusion data of the target K-feldspar through the multi-diffusion domain model (Lovera et al., 1989) and ^40^Ar/^39^Ar step heating experiment (Harrison and McDougall, 1982). However, the probable *t-T* path of the sampling site enables to constrain the hydrothermal K-feldspar closure temperature.

## 2 The Toki granite

The Toki granite in the Tono district, central Japan, is one of the Late Cretaceous plutonic bodies of the Sanyo Belt (Figure 1A). It is a stock, approximately 14 × 12 km^2^ in areal extent (Ishihara and Chappell, 2007; Ishihara and Suzuki, 1969), intruding into Jurassic sedimentary rocks of the Kamiaso unit in the Mino Belt and into the late Cretaceous Nohi Rhyolite (Itoigawa, 1980). The Toki granite is a zoned pluton with three rock facies grading from muscovite-biotite granite (MBG) at the margin through hornblende-biotite granite (HBG) to biotite granite (BG) in the interior (Yuguchi et al., 2010). Petrography and geochronology of the Toki granite were described in detail by Yuguchi et al. (2010; 2019B).

## 3 Sampling and analytical procedures

### 3.1 Sampling

#### 3.1.1 Sampling site and petrographical procedures

The Mizunami Underground Research Laboratory (Fig. 1B and C) has two vertical shafts (Main and Ventilation) approximately 500 m deep, ranging from an altitude of 201 m above sea level (masl) (ground level) to -299 masl (shaft bottom). This study employed borehole 06MI03 (336 m long), which was drilled vertically from an underground depth of 191 m, before continuing the excavation of the Ventilation Shaft below 191 m depth (Figure 1C). Yuguchi et al. (2019A) described the petrography, chemistry, and the mass transfer of plagioclase alteration for rock samples collected from the rock mass around 500 m depth (-274 to -314 masl) in the Ventilation Shaft (Figure 1C). A restricted sampling area ensures that samples followed the same temperature and pressure sub-solidus cooling history of the Toki granite (Yuguchi et al., 2019B). K-Ar geochronology for the hydrothermal K-feldspar was applied to sample 06MI03-8 (–304 masl), which is the same sample used for illite K-Ar dating by Yuguchi et al. (2019A). Petrography investigations of sample 06MI03-8 included backscattered electron (BSE) images and chemical maps collected using a JEOL IT100A scanning electron microscope with an energy-dispersive X-ray spectrometer (EDS) at Yamagata University, operating at an accelerating voltage of 15 kV and beam current of 1.5 nA.

#### 3.1.2 Separation and acquisition of hydrothermal K-feldspar from the rock sample

Separation of plagioclase from the rock sample followed by separation of hydrothermal K-feldspar from the plagioclase yielded a hydrothermal K-feldspar powder up to 60 μm in diameter and 0.278 g in weight following a two-step process. Two processes consisting of (1) the separation of plagioclase from the rock sample and then (2) the separation of hydrothermal K-feldspar from plagioclase were performed in accordance with (Sawai, 2006; Yagi, 2006; and Yagi and Itaya, 2011).

Step One: separation of plagioclase from the rock sample

(1)-1 Borehole core sample (06MI03-8) was cleaved into smaller pieces (rock chips roughly 1 cm^3^) and washed/dried.
(1)-2 The rock chips were crushed by a stamp mill and then washed by stirring in tap water and further processed by ultrasonic cleaning in deionized water to eliminate suspended particles.
(1)-3 Ferromagnetic minerals were eliminated from the dried fractions using a hand magnet. The residual ferromagnetic minerals and quartz were eliminated from the remaining fractions via an electromagnetic separator (Isodynamic® separator). The residual material consists of magmatic (magmatic origin) K-feldspar and plagioclase.
(1)-4 Magmatic K-feldspars were eliminated from the fractions through heavy liquid separation using a sodium polytungstate solution (heavy liquid of 2.60 g/cm^3^), leaving plagioclase grains because of the difference in density: orthoclase of 2.56 g/cm^3^, albite of 2.62 g/cm^3^, and anorthite of 2.76 g/cm^3^.

Step 2: the separation of hydrothermal K-feldspar from the plagioclase

(2)-1 The separated plagioclases were granulated into 60–100 μm grains through sieves. The plagioclase grains were treated with hydrochloric acid (30 min) at approximately 70 °C to eliminate small amounts of chlorite and smectite attached on the grain surface.
(2)-2 The plagioclase grains were washed five times with deionized water (approximately 70 °C) and then were placed in a beaker filled with deionized water inside an ultrasonic bath for 5 min to eliminate hydrochloric acid from the grain surface. Afterwards, the plagioclase grains were washed fifteen times with deionized water (approximately 70 °C) and dried.
(2)-3 The dried plagioclase grains were granulated again through sieves into smaller grains approximately 60 μm in diameter. The plagioclase grains were carefully pressed and dispersed in an agate mortar to separate the K-feldspar from the host plagioclase. The plagioclase grains were never crushed or milled in the mortar because such treatment could result in the loss of argon from the minerals.
(2)-4 Clerici solution is an aqueous mixture of equal parts of thallium formate [Tl(HCO_2_)] and thallium malonate [Tl(C_3_H_3_O_4_)], which is characterized by an easily controllable density ranging from 1.00 to 5.00 g/cm^3^ (Jahns, 1939). Clerici solution with a K-feldspar equivalent density of 2.56 g/cm^3^ allowed the separation between hydrothermal K-feldspar powders and plagioclase powders (albite: density of 2.62 g/cm^3^ and anorthite: density of 2.76 g/cm^3^). The centrifugation gathered the hydrothermal K-feldspar powders in the Clerici solution, and the target powders were collected. The resulting powders were washed with deionized water.
(2)-5 Desalination of the powders was conducted because the salty sample interferes with the high-precision determinations for potassium concentration and radiogenic argon (Nagao and Itaya, 1988). The powders were washed with deionized water (approximately 70 °C) by stirring in a beaker, which was placed in an isothermal bath at 70 °C for three days. The deionized water in the beaker was replaced 2–3 times a day, and aluminum foil was placed on the opening of the beaker to prevent slow evaporation. The washing could eliminate extremely-fine powders; the extremely-fine powders had potentially the loss of argon.
(2)-6 The desalination powders were dried and collected. The hydrothermal K-feldspar powders used for K-Ar geochronology had a weight of 0.278 g.

### 3.2 Analysis

#### 3.2.1 Analytical procedures for crystallography

The hydrothermal K-feldspar powders (0.01 g in the dating sample) were confirmed crystallographically using an X–ray powder diffractometer (XRD: Rigaku MiniFlex600) with a generation of 40 kV and 15 mA, 1.25° divergence slit, a 13 mm scatter slit, a 0.3 mm receiving slit, and a 2.5° solar slit. Profiles were collected between 2° and 40° 2θ using a step interval of 0.02° and a scanning rate of 20.0° per second. The plagioclases separated from the rock sample (0.01 g) were also identified. The powders employed in the XRD analysis were non-oriented. The mineral separation and XRD analysis were performed at the Hiruzen Institute for Geology and Chronology Co. Ltd.

#### 3.2.2 Quantitative analyses of radiogenic argon and potassium concentrations and age determination

Radiogenic argon concentrations for the K-Ar geochronology were measured with a 15 cm radius sector type mass spectrometer with a single collector system (HIRU housed at Research Institute of Natural Sciences, Okayama University of Science) with the isotopic dilution method and an ^38^Ar spike, which followed the analysis technique of Nagao and Itaya (1988) and Itaya et al. (1991). Radiogenic ^40^Ar was obtained from Eq. (1) (Itaya et al., 1991).

(1) [rad. ^40^Ar] = [sample ^40^Ar] – [air ^40^Ar] = [sample ^40^Ar] – 295.5 × [sample ^36^Ar] .

The ratio of air contamination in ^40^Ar (Air contamination/non-rad. ^40^Ar) was expressed as Eq. (2).

(2) {[sample ^40^Ar] – [rad. ^40^Ar]} / [sample ^40^Ar] × 100 (%) .

Potassium determination was carried out with a flame emission spectrophotometer (HITACHI 180-30 type at Research Institute of Natural Sciences, Okayama University of Science) utilizing an internal standard. Multiple analyses of the standard JG-1 biotite samples yielded an error of approximately 1% at a 2σ confidence level (Itaya et al., 1991; Yagi et al., 2015). The isotopic ages of the hydrothermal K-feldspar powders were calculated using the standard K-Ar age equation (Dalrymple and Lanphere, 1969; Nagao and Itaya, 1988; Dalrymple et al., 1999) with Eq. (3) of Steiger and Jäger (1977)

(3) λ_ε_ = 0.581 × 10^−10^ / yr, λ_β_ = 4.962 × 10^−10^ / yr and ^40^K / K = 1.167 × 10^−4^

The analytical uncertainties associated with the age determination were calculated using the method of Nagao and Itaya (1988).

## 4 Results

### 4.1 Petrography and crystallography

#### 4.1.1 Plagioclase alteration

**Figure 2:**
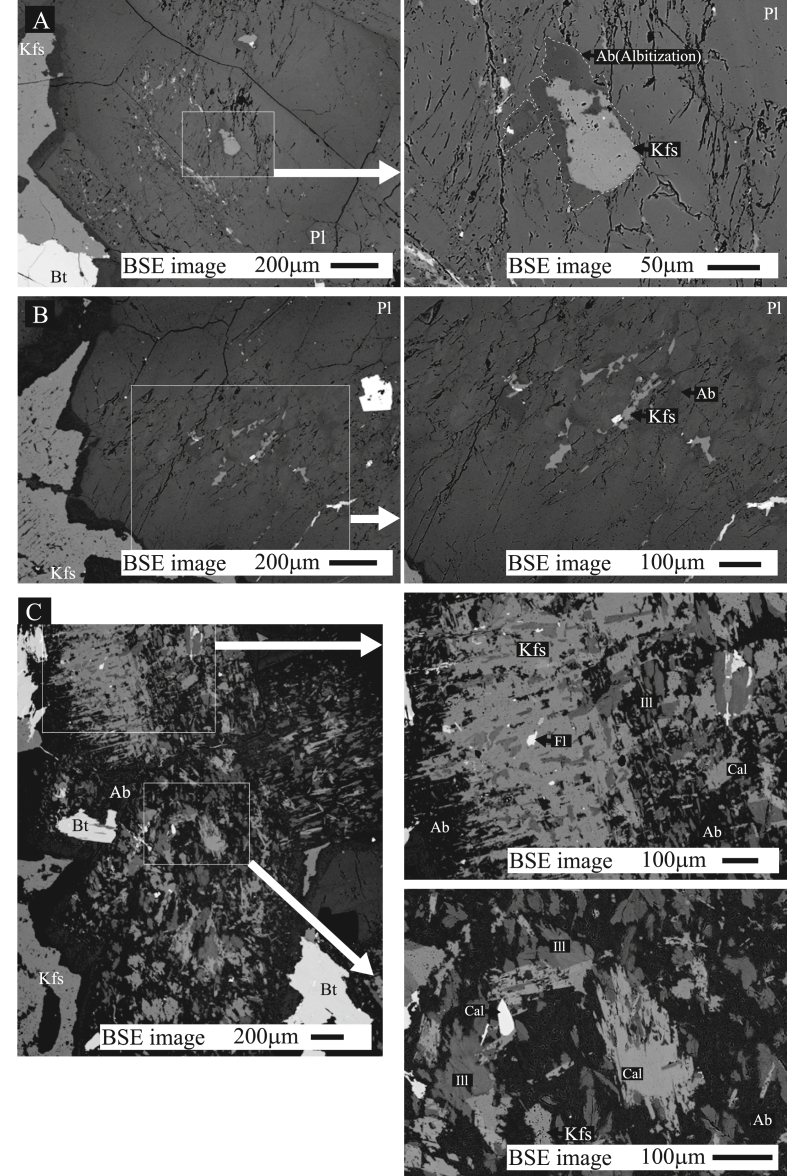
Backscattered electron (BSE) images of altered plagioclase. (A and B) solid-state replacement showing albite and dissolution–precipitation showing K-feldspar and albite, and K-feldspar, illite, calcite, and fluorite (C).

**Figure 3:**
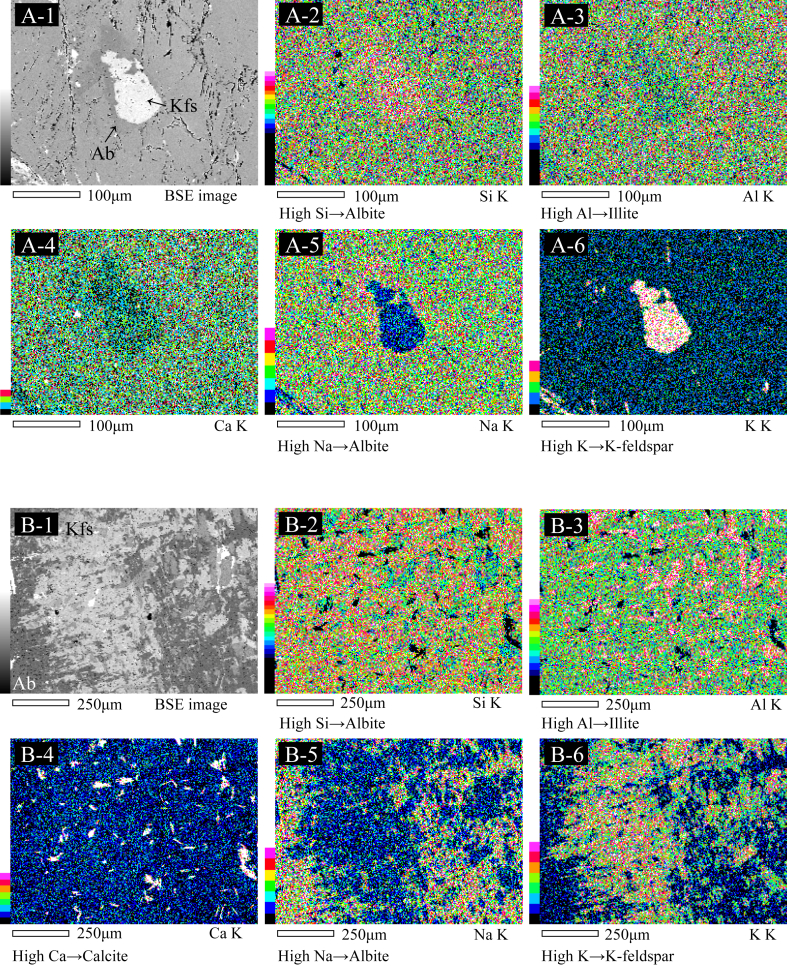
BSE images (A-1 and B-1) and chemical maps showing elemental Si, Al, Ca, Na, and K concentrations (A-2 to A-6 and B-2 to B-6) of samples No. 8. High concentrations are indicated by warm colors and low concentrations by cold colors. Illite, calcite, albite, and K-feldspar can be identified by the highest concentrations in elemental Al, Ca, Na, and K, respectively.

Plagioclase alteration, which is always accompanied by albitization and K-feldspathization and, additionally, by the formation of illite, calcite, and fluorite, is shown by BSE images (Figure 2) and chemical maps (Figure 3). Hydrothermal albite formed a matrix surrounding the K-feldspar in the altered plagioclase (Figure 2). The chemical composition of albite ranges from Ab_95_ to Ab_98_ (Figure 5 of Yuguchi et al., 2019A). K-feldspar occurs as variously sized patchy shapes from 10 to 400 μm wide (Figure 2) with a chemical composition of Or_87_ to Or_98_. Illite occurs as patchy shapes less than 80 μm wide, with irregular boundaries, corresponding compositionally to phengite (Yuguchi et al., 2019A). Calcite occurs as needle or columnar shapes less than 80 μm wide. Granular fluorite up to 20 μm is observed within the altered plagioclase.

#### 4.1.2 Crystallography

**Figure 4:**
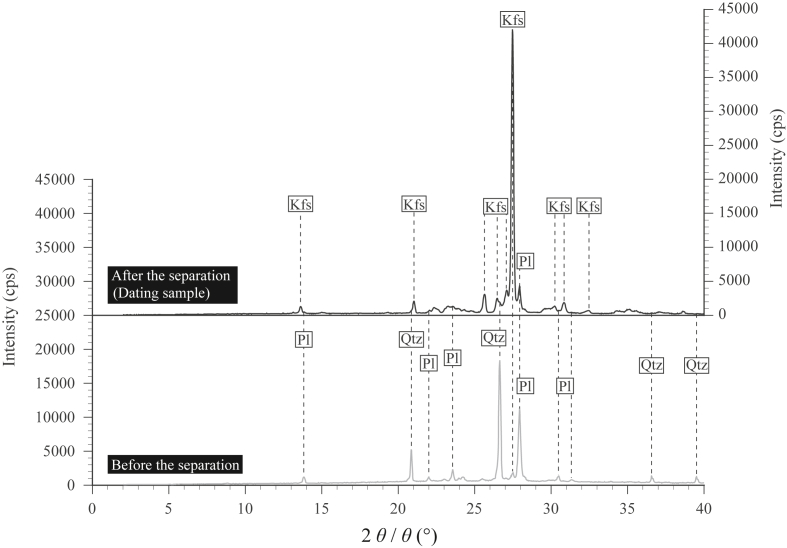
XRD patterns of the hydrothermal K-feldspar powders separated from plagioclase (0.01 g in the dating sample) and the plagioclase powders separated from a rock sample (0.01 g) of sample 06MI03-8. The occurrence of the microcline was identified crystallographically through the XRD pattern of the dating sample.

Figure 4 shows the XRD patterns of the plagioclase grains separated from rock sample 06MI03-8 (before K-feldspar separation) consisting of plagioclase, K-feldspar, and quartz where quartz is attached to the outer plagioclase surface, and hydrothermal K-feldspar powders separated from plagioclase (sample for dating) consisting of mostly microcline K-feldspar with minor plagioclase. The plagioclases comprise both albite-rich plagioclase from alteration and magmatic plagioclase, and they contain potassium below 0.6 wt.% (see Table 1 of Yuguchi et al., 2019A). Thus, the plagioclases do not influence the K-Ar geochronology results of K-feldspar. The XRD pattern of the dating sample also identifies the hydrothermal K-feldspar as microcline.

**Table 1:** K-Ar dating results for hydrothermal K-feldspar in the sample No. 06MI03-8.

Sample description					K (wt%)	Rad. ^40^Ar (10^-8^ cc STP/g)^3^	Non-Rad. ^40^Ar (%)	Age (Ma)	±	1σ
Borehole	Sample No.	Depth (mabh)^1^	Elevation (masl)^2^	Target mineral						
06MI03	8	505.0	-304.0	K-feldspar	10.767 ± 0.215	2645.1 ± 28.5	8.3	62.2	**±**	1.4

1 Depth from the ground surface denotes meters along borehole (mabh).

2 Altitude stands for meters above sea level (masl).

3 Volume of radiogenic 40 argon per unit mass (1 g) under the standard temperature (0 °C) and pressure (1 atm) conditions.

#### 4.1.3 Applicability to the hydrothermal K-feldspar in the K-Ar geochronology

Argon loss may occur via structural features, such as perthite lamellae and twin boundaries (Parsons et al., 1988). The hydrothermal microcline within the altered plagioclase does not show perthitic textures nor microcracks, although the magmatic K-feldspar does (Figure 2). The microcline does not show unusual twining patterns; therefore, we assume that the microcline had little argon-loss through the structural boundaries. The microcline in sample 06MI03-8 has not experienced tectonic deformation, indicating no structural transformation of microcline. The dating sample, therefore, provides a reasonable formation age for the microcline in the hydrothermal alteration.

### 4.2 Chronological results

Table 1 shows the results of the K-Ar dating for the hydrothermal K-feldspar powders separated from the plagioclase of rock sample 06MI03-8. The hydrothermal K-feldspar powders have potassium concentrations of 10.767 ± 0.215 wt.%; two values (10.7382 and 10.7963 wt.%) were averaged for the age calculation. Multiple quantitative analyses for the standard samples (JG-1 biotite) provide an error of about 1% of potassium concentration (Itaya et al., 1991; Yagi et al., 2015), and thus, the potassium concentration error for our sample is defined as 1%. The radiogenic ^40^Ar (rad. ^40^Ar) is 2645.1 ± 28.5 × 10^−8^ cc STP/g (per unit mass (1 g) at standard temperature (0 °C) and pressure (1 atm)). The determination of the potassium concentration and radiogenic ^40^Ar provides a K-Ar age of 62.2 ± 1.4 Ma for the hydrothermal K-feldspar.

When the K-Ar age of a hydrothermal K-feldspar is older than that of a magmatic K-feldspar, excess argon is potentially present in the hydrothermal K-feldspar. Thus, we compared the K-Ar age of the hydrothermal K-feldspar with that of the magmatic K-feldspar. The magmatic K-feldspar of sample MIU1-2 (-261.9 masl of borehole MIU-1) (Yuguchi et al., 2010), which was obtained near the samples of this study, has the K-Ar age of 68.2 ± 3.4 Ma (unpublished data collected by the Japan Atomic Energy Agency). That is, this sample is older than the hydrothermal K-feldspar (62.2 ± 1.4 Ma), indicating that the dating result of the hydrothermal K-feldspar is not influenced by excess argon.

## 5 Discussion

### 5.1 Thermal and temporal relationships between K-feldspathization and illitization

The plagioclase alteration process combined solid-state replacement and dissolution–precipitation processes (Yuguchi et al., 2019A). Illitization and K-feldspathization ages reflect dissolution–precipitation and solid-state replacement processes, respectively. Thermal and temporal relationships between K-feldspathization and illitization within plagioclase alteration lead to differences in formation conditions between the two processes.

**Figure 5:**
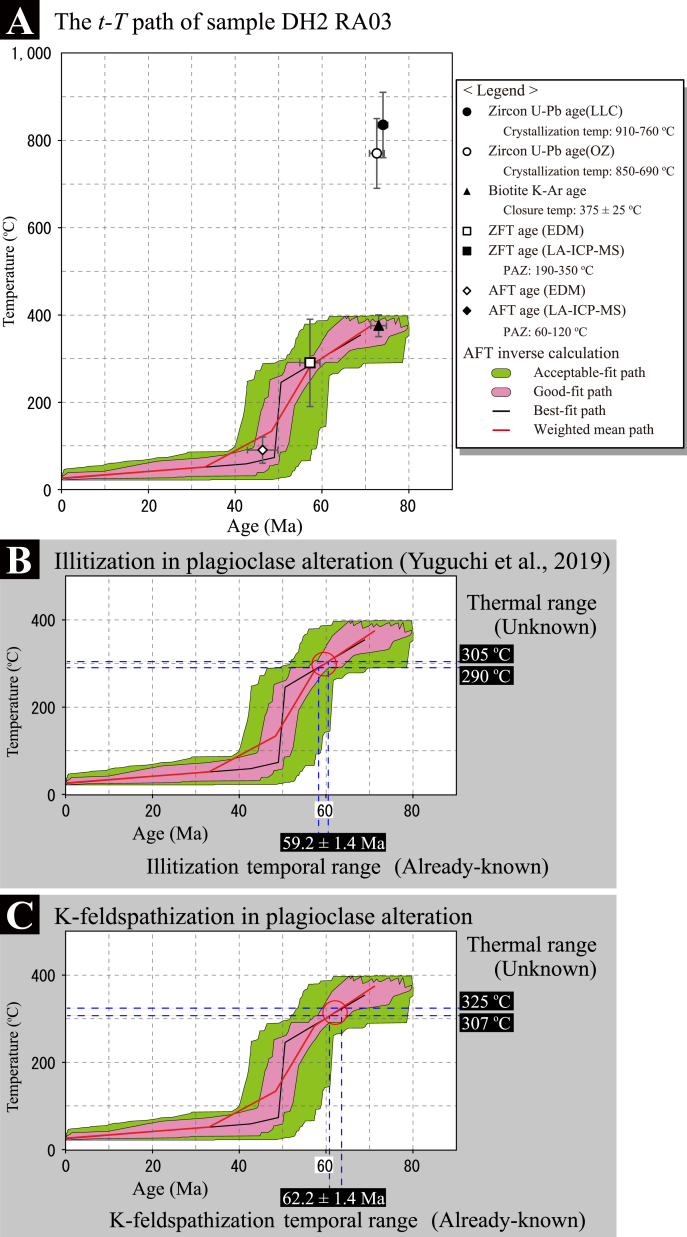
The time-temperature (*t-T*) path determining both the temporal and thermal conditions of the secondary minerals. (A) The *t-T* path of sample DH2 RA03 is constructed from thermochronological data, including zircon U–Pb ages, biotite K-Ar age, ZFT age, AFT age, and FT inverse calculation. The FT inverse model was calculated from the dataset including the AFT age, AFT lengths, ZFT age, and ZFT lengths, which provided the acceptable-fit paths, good-fit paths, best fit path, and weighted mean path below 400 °C. The envelope of good-fit paths include the biotite K-Ar, ZFT, and AFT ages, resulting in the reasonable reproduction of the *t-T* path from the biotite K-Ar closure temperature (350–400 °C) through the ZFT PAZ (190–390 °C) to the AFT PAZ (60–120 °C). (B) The unknown thermal condition for illitization during plagioclase alteration can be determined through the intersection of the weighted mean *t-T* path and the temporal condition of 59.2 ± 1.4 Ma (blue lateral lines), which provides a thermal range from about 305 °C to 290 °C for the dissolution–precipitation. (C) The unknown thermal condition for K-feldspathization during plagioclase alteration can be determined through the intersection of the weighted mean *t-T* path and the temporal condition of 62.2 ± 1.4 Ma (blue lateral lines), which provides a thermal range from about 325 °C to 307 °C for the solid-state replacement.

The K-feldspar and illite K-Ar geochronology of sample 06MI03-8 provide ages of 62.2 ± 1.4 Ma (Table 1) and 59.2 ± 1.4 Ma (Yuguchi et al., 2019A), respectively. The error range of the K-feldspar K-Ar age does not overlap with that of illite, and the K-feldspar K-Ar is older. The thermal condition of K-feldspar and illite in the plagioclase alteration was determined from its age using the *t-T* path of sample DH2 RA03 (-302.1 masl of borehole DH2) (Figure 5A), which was obtained nearby to this study's samples (approximately 90 m horizontally: Fig. S1D). Sample DH2 RA03 has a biotite K-Ar age of 73.0 ± 1.8 Ma (Yuguchi et al., 2011), a zircon fission-track (ZFT) age of 57.2 ± 2.3 Ma (Yuguchi et al., 2011), and an apatite fission-track (AFT) age of 46.3 ± 3.5 Ma (Yuguchi et al., 2019B). The closure temperature of a biotite K-Ar system is about 350–400 °C (Grove and Harrison, 1996), and the partial annealing zones (PAZ) of ZFT and AFT systems are 190–350 °C (Yamada et al., 2007; Bernet, 2009) and 60–120 °C (Green and Duddy, 1989; Corrigan, 1993), respectively. The K-Ar ages for magmatic and hydrothermal K-feldspars and illite lie between the biotite K-Ar age and the ZFT age of sample DH2 RA03. The *t-T* path of sample DH2 RA03 was constructed from the thermochronological data described in detail by Yuguchi et al. (2019B). The weighted mean *t-T* path (red path of Figure 5A) of the FT inverse model was employed for this discussion. The intersection of the *t-T* path and the K-feldspar K-Ar age of 62.2 ± 1.4 Ma (blue-dashed lateral lines) provides a range of 307–325 °C for the formation temperature (Figure 5B). Illitization occurred at the temporal conditions of 59.2 ± 1.4 Ma and temperature conditions of 290–305 °C (Yuguchi et al., 2019A: Figure 5C).

The temporal and thermal conditions of solid-state replacement (62.2 ± 1.4 Ma and 325–307 °C) are older and higher temperature than those of dissolution–precipitation (59.2 ± 1.4 Ma and 305–290 °C). Therefore, the plagioclase alteration within the granitic rock body includes serial processes, occurring from solid-state replacement to dissolution–precipitation, which is consistent with the petrological data of Yuguchi et al. (2019A).

### 5.2 Constraint on closure temperature of K-feldspar K-Ar geochronology

The closure temperature in the K-Ar system of hydrothermal K-feldspar is between 100 and 350 °C (Inger et al., 1996; McDougall and Harrison, 1999). Specifically, it ranges from 100 to 235 °C for the microcline in previous studies (Dodson and McClelland-Brown, 1985; Heizler et al., 1988; Lovera et al., 1989; Shibata, 1991; McDougall and Harrison, 1999). These closure temperatures were determined by ^40^Ar/^39^Ar step-heating experiments assuming a constant cooling of the pluton. For instance, Dodson and McClelland-Brown (1985) presented a closure temperature of 150 ± 30 °C in the microcline K-Ar system based on a constant cooling of 30 °C/myr for the Separation Point batholith. The multi-diffusion domain model (MDD) provides four pairs of closure temperatures and ages describing the *t-T* path for each K-feldspar sample, which leads to the K-feldspar closure temperature in the K-Ar system (Lovera et al., 1989). Heizler et al. (1988) obtained a closure temperature of 185 ± 6 °C in the microcline in the K-Ar system, and a revised study for the same sample by Lovera et al. (1989) provides a temperature of 156–235 °C based on MDD. These discussions indicate that more reliable *t-T* paths for rock samples are significant in determining greater closure temperature accuracy.

The Toki granite has position-specific *t-T* paths (Yuguchi et al., 2019B). The probable path of sample DH2 RA03 provides closure temperature constraints of the hydrothermal microcline. The intersection of the *t-T* path and the 62.2 ± 1.4 Ma microcline K-Ar age provides a formation temperature of 307–325 °C (Figure 5B), which corresponds to the microcline K-Ar system closure temperature. Our case study indicates that the closure temperature in this system is higher than the temperatures of 100–235 °C presented by previous studies, suggesting that a combination of microcline K-Ar dating and an accurate *t-T* path for the sampling site within the granite can determine the distinct closure temperature in the K-Ar system.

## 6 Conclusions

Our methodology and interpretations provide new insight for K-Ar geochronology in hydrothermal microcline within altered plagioclase in a granitic pluton. Our methodology employs a two-step separation process consisting of 1) plagioclase extraction from the rock sample and 2) separation of the hydrothermal microcline from the plagioclase, giving precise determination of microcline powders in K-Ar geochronology. In our interpretation, the combination of the microcline K-Ar age of 62.2 ± 1.4 Ma and the accurate *t-T* path (thermochronology for multiple minerals) for the granite sampling site limits the thermal conditions (307–325 °C) for hydrothermal alteration phenomena, enabling to constrain the microcline K-Ar system closure temperature. This tighter constraint should provide the ability to better unravel thermal and age histories in granite subject to multi-step alteration processes and complex thermal histories.

## Declarations

### Author contribution statement

Takashi Yuguchi: Conceived and designed the experiments; Performed the experiments; Analyzed and interpreted the data; Contributed reagents, materials, analysis tools or data; Wrote the paper.

Koshi Yagi: Performed the experiments.

Eiji Sasao: Contributed reagents, materials, analysis tools or data.

Tadao Nishiyama: Analyzed and interpreted the data; Wrote the paper.

### Funding statement

Financial support came from the 10.13039/501100001691Japan Society for the Promotion of Science (JSPS) KAKENHI for Young Scientists [grant number 16H06138] and the 10.13039/501100003050Ministry of Economy, Trade and Industry (METI), Japan, grant to TY.

### Competing interest statement

The authors declare no conflict of interest.

### Additional information

No additional information is available for this paper.
